# Cat scratches, not bites, are associated with unipolar depression - cross-sectional study

**DOI:** 10.1186/s13071-015-1290-7

**Published:** 2016-01-05

**Authors:** Jaroslav Flegr, Zdeněk Hodný

**Affiliations:** Department of Biology, Faculty of Science, Charles University in Prague, Viničná 7, 128 44 Prague, Czech Republic; Department of Genome Integrity, Institute of Molecular Genetics ASCR, v.v.i, Prague, Czech Republic

**Keywords:** Toxoplasmosis, Unipolar depression, Injury, Major depression, Parasite, Bartonelosis

## Abstract

**Background:**

A recent study performed on 1.3 million patients showed a strong association between being bitten by a cat and probability of being diagnosed with depression. Authors suggested that infection with cat parasite *Toxoplasma* could be the reason for this association.

**Method:**

A cross sectional internet study on a non-clinical population of 5,535 subjects was undertaken.

**Results:**

The subjects that reported having been bitten by a dog and a cat or scratched by a cat have higher Beck depression score. They were more likely to have visited psychiatrists, psychotherapists and neurologists in past two years, to have been previously diagnosed with depression (but not with bipolar disorder). Multivariate analysis of models with cat biting, cat scratching, toxoplasmosis, the number of cats at home, and the age of subjects as independent variables showed that only cat scratching had positive effect on depression (*p* = 0.004). Cat biting and toxoplasmosis had no effect on the depression, and the number of cats at home had a negative effect on depression (*p* = 0.021).

**Conclusions:**

Absence of association between toxoplasmosis and depression and five times stronger association of depression with cat scratching than with cat biting suggests that the pathogen responsible for mood disorders in animals-injured subjects is probably not the protozoon *Toxoplasma gondii* but another organism; possibly the agent of cat-scratched disease – the bacteria *Bartonella henselae*.

## Background

Recently, an exploratory study performed with a data mining technique on electronic records of 1.3 million patients of the University of Michigan Health System showed the existence of a strong association between dog and cat-bite injuries and the probability of being diagnosed with depression at some point in life [1]. The association was stronger for cat bites than dog bites. While only 9 % of all patients of the data set were ever diagnosed with depression, this diagnosis (mostly ICD 311, ICD 296.30, ICD 296.20) was found in 41.3 % of those with cat bites and 28.7 % of those with dog bites. The probability of depression disorder in cat-bitten subjects was stronger for women (47.0 %) than for men (24.2 %). Animal bites preceded being diagnosed with depression in 27 % and 36 % patients bitten by a dog and a cat, respectively.

The authors suggested that infection with *Toxoplasma gondii* could play a role in the observed association. This protozoan parasite has a cat as definitive host, any homoeothermic vertebrate as intermediate or paratenic hosts, and can infect humans by several routes [2]. The oocystes of the parasite contained in feces of infected cats can survive in soil for many months; therefore, human infection can occur not only by direct contact with soil or consumption of unwashed vegetables, but also by soil on the paws and fur of both dogs and outdoor cats. Several studies have shown that *Toxoplasma* – infected subjects have higher levels of depression [3], anxiety [4] and a higher risk of suicide [5, 6]. It was suggested that decreased concentration of tryptophan and consequently serotonin – a part of the general defense of an organism against various infections – could be the reason for depression and possibly also for the increased rate of suicide in *Toxoplasma*-infected subjects [7]. However, cat and dog bites are not considered to be a source of *Toxoplasma* infection, and no data indicate that the bitten subjects in the population of Michigan patients are in fact *Toxoplasma* infected.

To verify the result of the exploratory analysis of the Michigan group and to test the *Toxoplasma* hypothesis of depression in bitten patients, we performed a large cross sectional study on non-clinical population of subjects tested for the presence of anamnestic titres of anti-*Toxoplasma* antibodies. Using an electronic questionnaire we measured the depression scores of responders, and using an anamnestic questionnaire we monitored the intensity of their current and past contacts with cats and dogs. We also asked the responders whether they have ever been bitten by a dog, bitten or scratched by a cat, whether they attended psychiatry or neurology in past two years, whether they suffer any neurological and mental health problems, whether they were taking antidepressants at the time of the study, and whether they had been diagnosed with unipolar depression or bipolar disorder. We analyzed the existence of the reported association between animals bites and depression in the normal population, whether it concerned only cat bites or also cat scratches, and whether it was still detectable when the confounding variables of the number of cats and dogs in a household and *Toxoplasma* infection were statistically controlled.

## Methods

### Participants and procedure

The subjects were invited to participate in the study using a Facebook-based snowball method [8], namely by posting an invitation to participate in “an experiment searching for associations between keeping dogs and cats and health status and personality of subject” on the wall of the Facebook page “Guinea Pigs” for Czech and Slovak nationals willing to take part in diverse evolutionary psychological experiments (www.facebook.com/pokusnikralici). This Facebook community consists of people of various ages, education levels, occupations and places of living. They are not compensated for participation in studies, however, they are being regularly informed about results of studies and about news in science and psychology on the Guinea Pigs web page. The former students of biology who were tested for toxoplasmosis during systematic research of behavioral effects of latent toxoplasmosis which has been running at the Faculty of Science in past years were probably overrepresented among the subjects who knew their *Toxoplasma*-infection status. The participants were informed about the aims of the study on the first page of the electronic questionnaire. They were also provided with the following information: “The questionnaire is anonymous and obtained data will be used exclusively for scientific purposes. Your cooperation in the project is voluntary and you can terminate it at any time by closing this web page. Please share the link to this questionnaire with your friends, for example on Facebook”. The share button was pressed by 840 participants, which resulted in obtaining data from 6007 responders in total between 20th August 2014 - 7th January 2015.

### Ethic consents and permissions

All participants provided informed consent by pressing corresponding button at the electronic form. The study including the method of acquiring electronic informed content was approved (No. 52013/07) by the IRB of the Faculty of Science, Charles University in Prague, and was conducted in accordance with the Helsinki Declaration as revised 1989.

### Electronic questionnaire

The questionnaire was distributed as a Qualtrics survey. It contained a standard Czech version of the Five Item Personality Inventory (FIPI) [9], Beck Depression Inventory [10], Obsessive-Compulsive Inventory – Revised (OCI-R) [11], Toxo1 questionnaire [12] and anamnestic questionnaire containing, among other things, questions about the intensity of contact with dogs and cats and about animal-related injuries. Subjects were asked to rate the intensity of their life-long contact with dogs and cats using the 7-step scale: (1: never, 2: we kept a dog (cat) only in past and only for a short time, 3: we kept a dog (cat) only in past but for a long time, 4: we have one dog (cat), 5: we have two dogs (cats), 6: we have three dogs (cats), 7: we have more than three dogs (cats). They were also asked to rate intensity of sustained animal-related injuries using the following scale: 1- never, 2- just in play, 3- yes but just to warn me, 4- yes but not seriously, 5- yes, seriously, 6- yes very seriously, I had to seek for medical help, 7- I was seriously bitten (scratched) by several dogs (cats). Probands were asked to check the variant describing the most serious injury they suffered. Before the analyses, the responses concerning animal-related injuries were recoded to binary variables 0 (1 and 2), 1 (3–7) to be able to reproduce analysis performed in previous study [1], however, the results of analyses that used the unreduced scale were approximately same. The subjects were also asked whether they are *Toxoplasma* infected. They were reminded that *Toxoplasma* is “a parasite of cats, dangerous especially to pregnant women”. Implicitly, the response “I do not know, I am not sure” was selected. Responders could change this by selecting either “No, I was tested by a doctor and the result of my laboratory tests was negative” or “Yes, I was tested by a doctor and I had antibodies against *Toxoplasma*”. At the very end of the questionnaire, the participants were given an opportunity to sign the questionnaire by their member code. About 3 % of responders have done this and 18 of them have provided the information about their *Toxoplasma*-infection status. We found that all of them had provided correct information about their status. Participants were also asked to rate whether they suffered any neurological or mental health problems (1: not at all, 7: many or serious), to select which mental health problem they had been diagnosed with from a list of 20 mental health disorders, to select which medical doctors they visited in past 2 years from a list of 25 specialists, and which prescription medical drugs they were taking regularly from a list of 30 categories of drugs. The questionnaire took subjects 20–40 minutes to complete and about 72 % of subjects who started the study finished the whole questionnaire.

### Data analysis

Before statistical analysis, suspicious data (too high or too low values for height, body mass, age or duration of the test etc.) were filtered out (30 subjects). In the test, we also monitored personality profiles and many health status related variables. However, here we have analyzed only data concerning depression.

SPSS v. 21. was used for all statistical tests. Two-sided tests were used in all analyses. Ordinal and binary data were analyzed by partial Kendall’s correlation test [13, 14]. This test measures the strength and significance of the association between binary, ordinal, and continuous data, regardless of their distributions. This technique enables controlling for one confounding variable, for example, the age of a responder. The Excel sheet for computing partial Kendall’s Tau and the significance between variables A and B after the variable C is controlled, based on Kendall Tau’s AB, AC and BC, is available here: http://web.natur.cuni.cz/flegr/programy.php. The model derived residuals for depression score had approximately normal distribution. Therefore, we used multivariate analysis of covariance ANCOVA to search for effects of the binary variables of cat-related injury and *Toxoplasma* seropositivity, the continuous variable of the age of subjects, and the ordinal variables of the number of cats and dogs at home, on depression. The relationship between two binary variables, namely between animal-related injury and *Toxoplasma* seropositivity status, was studied with contingency tables. Many studies have shown that *Toxoplasma* infection has a different impact on men and women [15–17]. Similarly, the original study showed that the association between cat bites and depression disorder was stronger in women. Therefore, we also performed all analyses separately for male and female responders.

## Results

### Are animal-related injuries associated with depression?

**Fig. 1:**
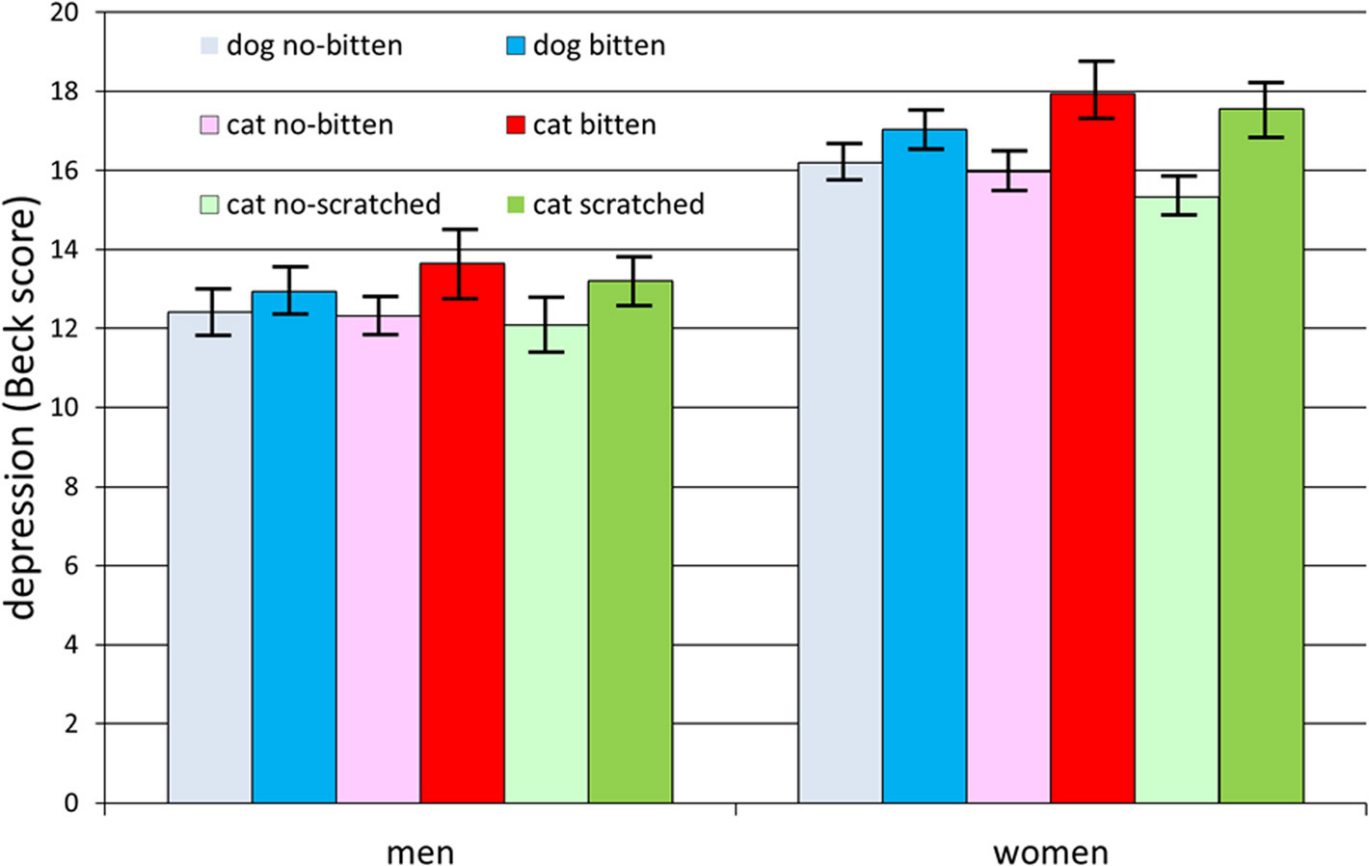
Effects of a dog biting and a cat biting/scratching on Beck depression score. *Hights of columns shows awerage Back depression score in particular groups, the spreads show 95 % C.I*

Final data set contained records of 2,064 men and 3,471 women. Men were significantly older (34.2, SD = 11.9) than women (31.5, SD = 11.5), *p* < 0.0001. In men who responded to the questions about animal-related injuries, 778 (43.6 %) had been bitten by a dog, 447 (25.5 %) had been bitten by a cat, and 886 (51.3 %) had been scratched by a cat. In women who responded these questions, 1,266 (40.4 %) had been bitten by a dog, 922 (29.8 %) by a cat, and 1,670 (54.3 %) had been scratched by a cat. The Beck depression score of subjects bitten/scratched by a dog or a cat was higher than that of non-bitten/non-scratched controls, Fig. 1. The depression score decreased with age both in men (*p* < 0.001, R^2^ = 0.01) and women (*p* < 0.001, R^2^ = 0.01). The subjects bitten/scratched by animals, especially women bitten/scratched by a cat, also expressed other indications of depression and impaired mental health. They were more likely to have visited neurologists, psychiatrists and psychotherapists in the past two years, had been taking more antidepressants, reported more mental health and neurological problems, and were more likely to have been diagnosed with unipolar depression (but not bipolar disorder), Table 1.

**Table 1:** Indices of various depression problems in men and women bitten by dog or bitten/scratched by a cat

	Dog bitten		Cat bitten		Cat scratched	
			Women			
	Tau	p	Tau	p	Tau	p
Attended neurology	−0.008	0.578	0.022	0.112	0.037	**0.006**
Attended psychiatry	0.028	**0.041**	0.074	**0.000**	0.059	**0.000**
Attended psychotherapy	0.032	**0.019**	0.034	**0.012**	−0.001	0.950
Taking antidepressants	−0.001	0.929	0.014	0.302	0.031	**0.019**
Neurological problems	0.058	**0.000**	0.047	**0.000**	0.046	**0.000**
Mental health problems	0.054	**0.000**	0.092	**0.000**	0.091	**0.000**
Unipolar depression	0.014	0.285	0.027	**0.041**	0.046	**0.000**
Bipolar disorder	0.026	**0.048**	0.008	0.536	0.010	0.464
Beck depression score	0.040	**0.002**	0.062	**0.000**	0.081	**0.000**
			Men			
	Tau	p	Tau	p	Tau	p
Attended neurology	0.060	**0.001**	−0.023	0.220	−0.015	0.438
Attended psychiatry	−0.010	0.592	0.033	**0.078**	0.036	**0.054**
Attended psychotherapy	0.055	**0.004**	0.001	0.953	0.070	**0.000**
Taking antidepressants	−0.046	**0.012**	0.025	0.168	0.020	0.279
Neurological problems	0.085	**0.000**	0.017	0.350	0.073	**0.000**
Mental health problems	0.049	**0.008**	0.092	**0.000**	0.042	**0.021**
Unipolar depression	0.002	0.924	0.050	**0.006**	0.018	0.334
Bipolar disorder	0.008	0.660	−0.015	0.415	−0.003	0.873
Beck depression score	0.046	**0.009**	0.056	**0.001**	0.068	**0.000**

The table shows results of partial Kendall analysis with age of subjects as covariate. Significant results and trends (*p* < 0.1) are printed in bold. Positive Kendall Tau means positive relation between the variables. *P* value 0.000 means *P* < 0.001. No correction for multiple tests were performed, however, theoretical number of false positive tests should be 2–3, not 33

### Are animal-related injuries related with depression in subjects non-taking antidepressants?

The separate univariate ANCOVA analyses performed on subjects taking and not taking antidepressants showed nonsignificant associations of animal bites with depression in the former group (men: dog biting *p* = 0.267, eta^2^ = 0.036, cat biting *p* = 0.627, eta^2^ = 0.007, cat scratching *p* = 0.563, eta^2^ = 0.01; women: dog biting *p* = 0.460, eta^2^ = 0.004, cat biting: *p* = 0.327, eta^2^ = 0.006, cat scratching *p* = 0.126, eta^2^ = 0.016) and significant associations in the latter group (men: dog biting *p* = 0.004, eta^2^ = 0.006, cat biting *p* = 0.009, eta^2^ = 0.005, cat scratching *p* = 0.036, eta^2^ = 0.004; women: dog biting *p* = 0.045, eta^2^ = 0.045, cat biting: *p* < 0.001, eta^2^ = 0.008, cat scratching *p* < 0.001, eta^2^ = 0.010). The effect sizes were similar in both groups and no significant injury-antidepressant interaction was detected by bivariate ANCOVA.

### *Does* Toxoplasma *infection play a role in the association between animal-injuries and depression?*

**Fig. 2:**
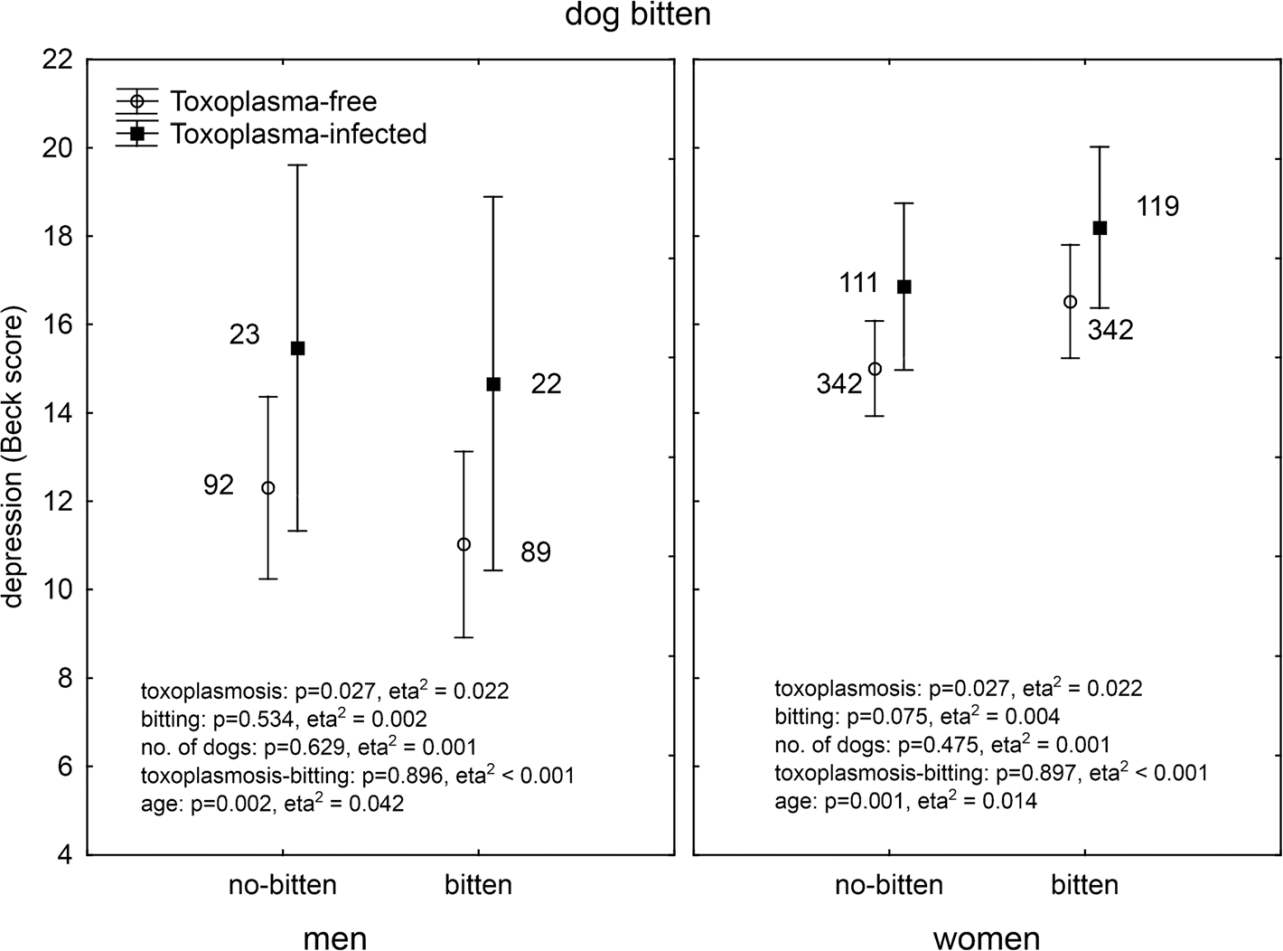
Effect of dog biting and toxoplasmosis on depression. *The results were obtained with ANCOVA with age as a covariate. Means with 95 % C.I. and number of subject in particular group are shown*

It was suggested by authors of the original study that *Toxoplasma gondii*, the parasite of cats, could play a role in cat bite-associated depression. Of our responders, 1,042 (19 %) had been serologically tested for toxoplasmosis and reported the result of their test. In men, 206 reported negative and 55 (21.1 %) positive results while in women 620 reported negative and 243 (28.2 %) positive results (Chi^2^ = 5.16, *p* = 0.023). ANCOVA analysis showed a significant association between toxoplasmosis and depression in both men (*p* = 0.021, eta^2^ = 0.023) and women (*p* = 0.021, eta^2^ = 0.007). This suggests that the biting/scratching by a cat could be just a proxy for close contacts with cats and therefore higher probability of being *Toxoplasma* infected (and suffering *Toxoplasma*-associated depression). To test this hypothesis, we performed multivariate analysis of covariance with depression as the dependent variable, binary variables of *Toxoplasma* infection and dog or cat bites or cat scratches, and continuous variables of the age of subjects and ordinal variable of the number of dogs (or cats) in the subject’s household as independent variables. The analyses were performed separately for men and women. A comparison of the Figs. 2 (dog bites), 3 (cat bites), 4 (cat scratches) indicates that this hypothesis could be true for dog bites. Here, the biting had no effect on depression while toxoplasmosis had a significant effect on depression both in men and women.

**Fig. 3:**
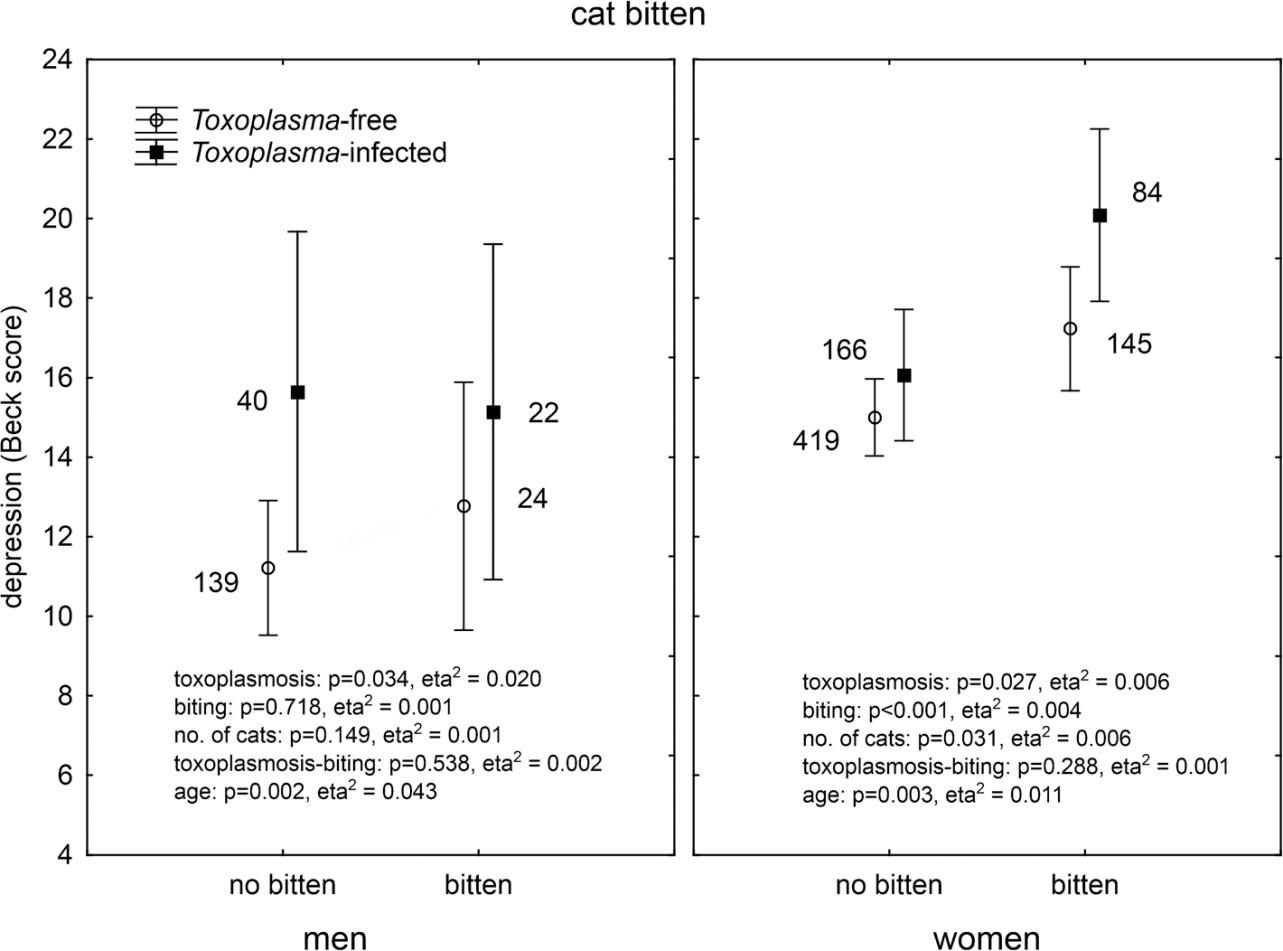
Effect of cat biting and toxoplasmosis on depression. *The results were obtained with ANCOVA with age as a covariate. Means with 95 % C.I. and number of subject in particular group are shown*

**Fig. 4:**
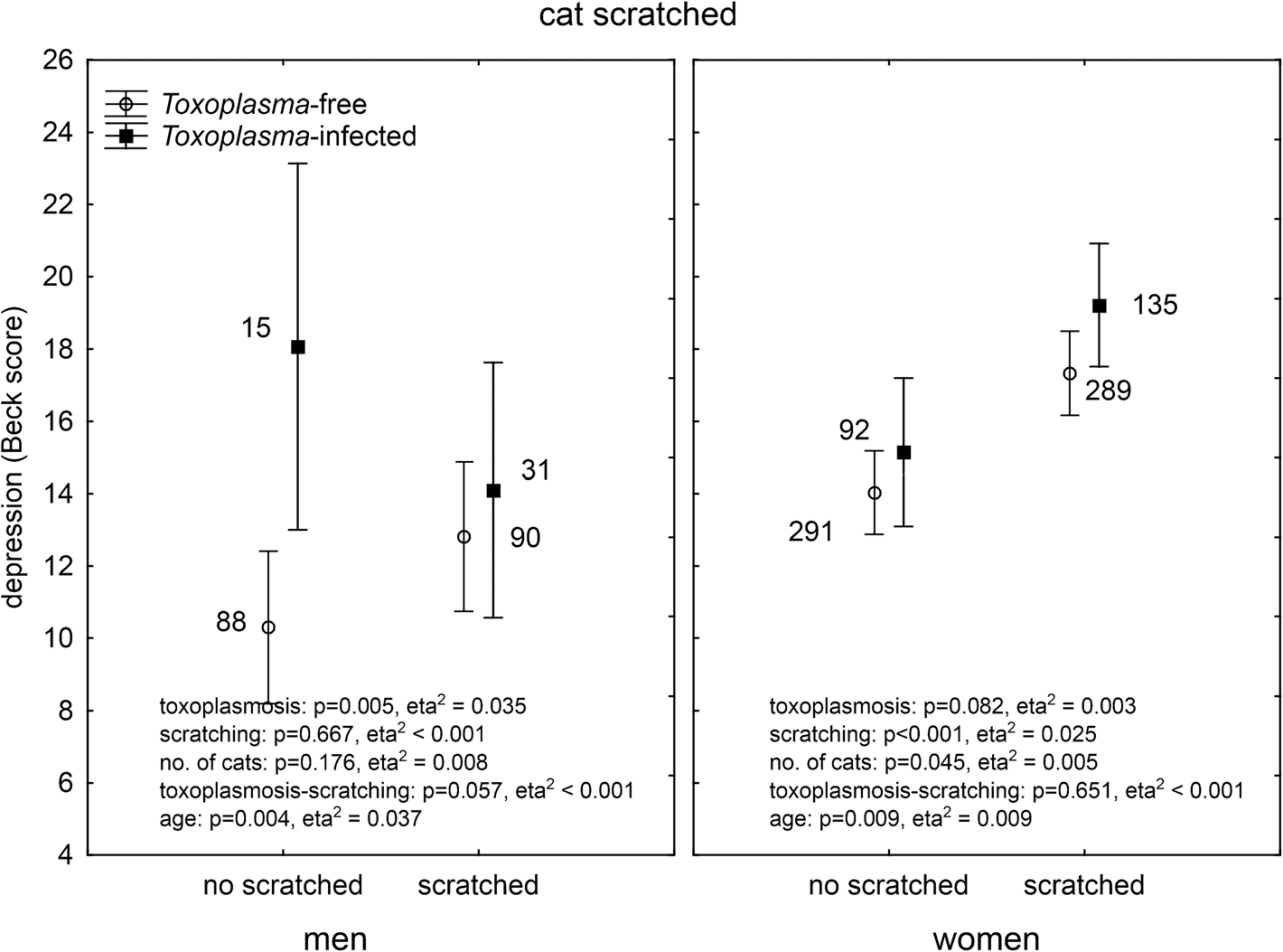
Effect of cat scratching and toxoplasmosis on depression. *The results were obtained with ANCOVA with age as a covariate. Means with 95 % C.I. and number of subject in particular group are shown*

The situation was more complicated with the cat biting and scratching. The association between toxoplasmosis and depression was significant in both men and women. However, in women, highly significant effects of biting, and especially scratching (more than 5 times larger effect size of cat scratching), were also observed.

### What kind of animal-related injury plays primary role in the injury-depression association?

**Fig. 5:**
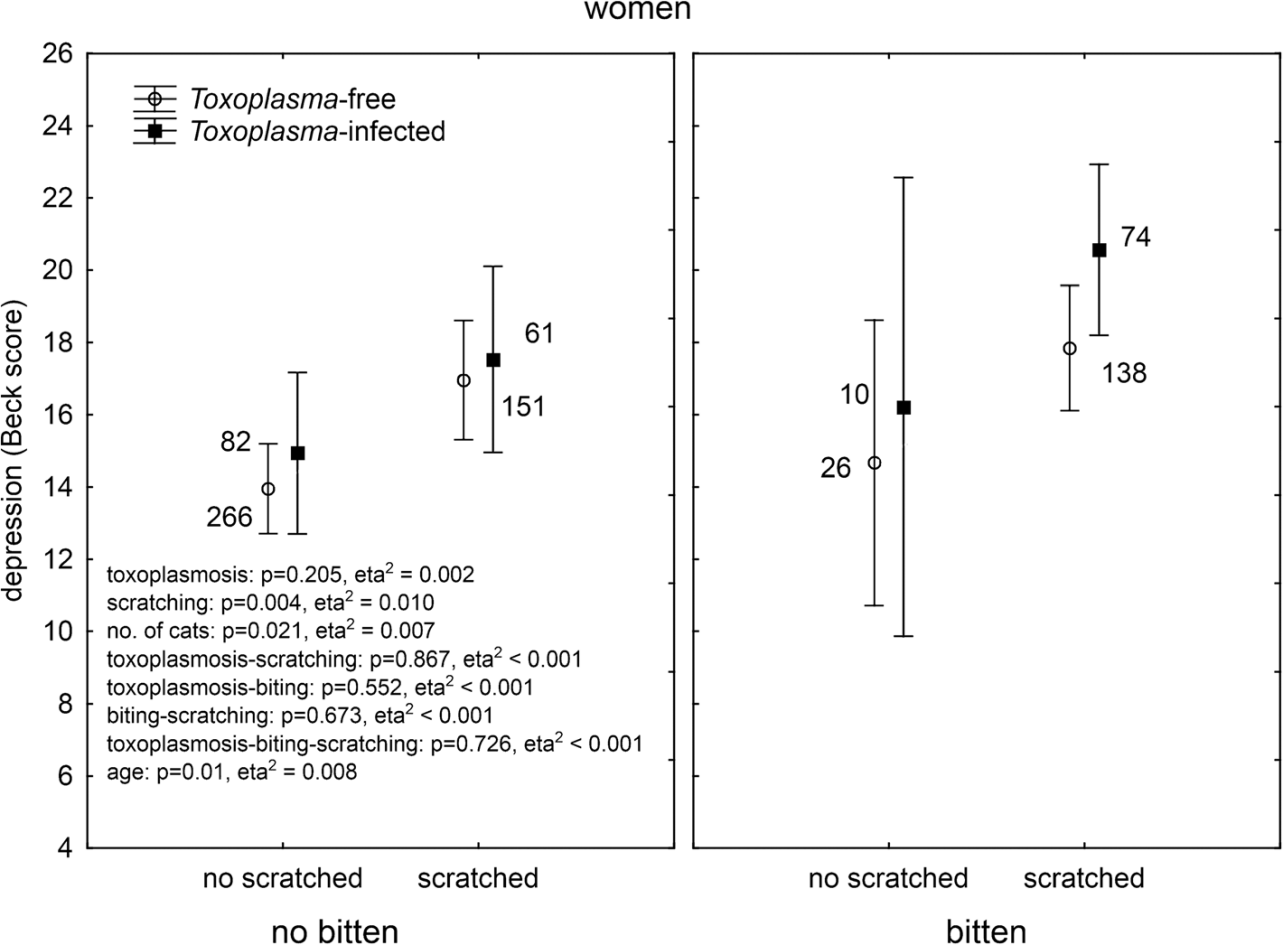
Effect of cat biting, cat scratching and toxoplasmosis on depression. *The results were obtained with multivariate ANCOVA with age as a covariate. Means with 95 % C.I. and number of subject in particular group are shown*

The analysis of models containing both cat bites and cat scratches (and toxoplasmosis, number of animals in the household, and age) as independent variables showed no significant effect for men, except a weak ternary interaction, toxoplasmosis-cat biting-cat scratching (*p* = 0.031, eta^2^ = 0.001). This result, however, was highly unstable as only one *Toxoplasma*-infected man bitten by a cat but non-scratched by a cat was present in our large data set. The results of this analysis performed in women is shown in Fig. 5. The analysis strongly indicated that neither toxoplasmosis nor the cat biting, but rather the cat scratching could be responsible for increased depression in women. The second significant effect was the effect of the number of cats in the household. It is important to stress, however, that the number of cats had a negative effect on depression (the more cats at home, the less depressed the women were) when the confounding variables (*Toxoplasma*, age, cat biting and cat scratching) were controlled.

### Could animal-related injuries play a role in transmission of toxoplasmosis?

Transmission of toxoplasmosis by cat/dog biting or scratching has never been reported. However, number of subjects in particular categories (see Figs. 3 and 4) suggests the existence of the association between cat bites/scratches and the *Toxoplasma* infection. Contingency tables analysis indeed showed a significant association between toxoplasmosis and cat biting/scratching both in men (biting: *p* < 0.001, scratching: *p* = 0.034) and women (biting: *p* = 0.042, scratching: *p* = 0.017). The association with dog biting was absent in men (*p* = 0.809) but present in women (*p* = 0.008).

## Discussion

Our cross sectional study confirmed the association between being bitten by a cat and suffering depression. We showed that the bitten subjects, especially women, expressed higher depression scores in the Beck Depression Inventory, had visited psychiatrists, psychotherapists and neurologists in past two years more often, were more likely to be using antidepressants, reported more neurological and mental health problems, and had been more often diagnosed with unipolar depression. We also confirmed the existence of already reported association between depression and presence of anamnestic titres of anti-*Toxoplasma* antibodies. More detailed analysis, however, showed that neither cat bites nor toxoplasmosis, but rather cat scratches are primarily associated with depression in women. For the first time, we demonstrated the association between being bitten by a cat or a dog (in women only) and presence of anti-*Toxoplasma* antibodies.

In the original study, the association between cat biting and depression was recognized by a data mining approach on clinical data of 1.3 million patients. In this population only 750 (0.05 %) subjects had been bitten by a cat and 1,108 (0.09 %) by a dog. These small subpopulations of people that seek medical help after the cat or dog-related injury may be rather atypical, which could explain their depression-related diagnoses. Our study was performed on a general population. In fact, our results suggest that a large fraction of normal people have been bitten by a dog (46 % men and 40 % women) and a cat (25 % men and 30 % women) or scratched by a cat (51 % men and 54 % women) during their life. Fraction of subjects who reported to be “seriously bitten by a cat” was much lower, namely 0.9 % in our population. We observed the association between animal-related injuries and (mostly subclinical) depression measured with the Beck Depression Inventory in subjects not taking antidepressants. It suggests that the association also occurs in subjects without clinical depression.

We found a significant effect of being bitten by a cat or a dog on the probability of being *Toxoplasma* seropositive. Animal-related injuries are not considered to be an epidemiological risk factor for toxoplasmosis [18, 19]. This infection is usually acquired by ingestion of food or water contaminated with cat feces containing *Toxoplasma* oocystes or by ingestion of tissue cysts contained in a raw or undercooked meat of intermediate hosts, e.g. pigs or sheep. There are also recent indications that toxoplasmosis can be transmitted from men to women during sexual intercourse and that this kind of transmission (as well as serial congenital transmission) definitively occurs in other animal species [20]. *Toxoplasma* is very universal and well adapted parasite, therefore a possible role of animal-related injuries in its transmission cannot be rejected off-hand and should be a subject of future epidemiological studies.

The primary role of cat scratching and no effect of toxoplasmosis seropositivity observed in our study indicate that not *Toxoplasma*, but rather another pathogen could be responsible for the reported phenomena. We hypothesize that this pathogen could be bacteria *Bartonella*, most likely *Bartonella henselae*, This gram-negative bacterium is present in the blood and other body fluids and tissues of 50 % cats in many regions through the world [21]. It causes the relatively common cat-scratch disease as well as several other less frequent but more serious diseases such as bacillary angiomatosis, infectious endocarditis, and Oroya fever [22]. *Bartonella henselae* is transmitted from cats to humans through cat scratches, mostly by contamination of scars by bacteria-containing flea feces. It can be also transmitted by a cat bite, by flea bite, and possibly also by ticks. Several other species of the genus *Bartonella* are present also in cats and other hosts [23]. Cat-scratch disease was described relatively recently, but its frequency could in fact be high. Seroprevalence of anti-*Bartonella* antibodies in the human population was estimated at 5-30 % and incidence of (recognized and reported) cases of *Bartonella* infections in the USA was 9.3 per 100 000 inhabitants in 1993 [24]. Infected subjects, mostly children, usually develop skin lesions and unilateral lymphadenitis in the lymph-draining region of the site of injury. Patients can suffer of low grade fever, aching, malaise, anorexia, headache, or splenomegaly. Typically, the disease is self-limiting and patients do not require any treatment. Sometimes, however, the lymphadenopathy persists several months and more serious sequels can occur, including encephalopathy, neuroretinitis, or osteomyelitis. Relatively often, neurological symptoms of the infection develop, such as severe headache, acute confusion, seizures, and focal neurological deficits [25, 26]. A recent metaanalytic study has showed that no effective method of treatment of cat-scratch disease is currently available (Prutsky 2013).

Toxoplasmosis was reported to be associated with specific behavioral changes in infected animals, and behavioral and personality changes, including serious mental health disorders, in humans. It was, however, already suggested that *Toxoplasma* infection could be just an indicator of probable contact with another pathogen transmitted to human from domestic cats [12]. This suggestion was based on the fact that in some studies on association between toxoplasmosis and schizophrenia, contact with a cat, rather than *Toxoplasma* seropositivity, was associated with increased risk of schizophrenia [27, 28]. It must be admitted, however, the fact that genes for key enzymes involved in the synthesis of dopamine exist in the genome of *Toxoplasma* [29], their expression in the brain of the infected host [30], as well as the presence of morphological changes (decreases in gray matter density bilaterally in the caudate, median cingulate, thalamus, occipital cortex, and in the left cerebellar hemispheres) exclusively in brain of *Toxoplasma*-infected schizophrenia patients [31], strongly suggests that *Toxoplasma* plays a principal role in the etiology of certain forms of schizophrenia [32]. This may be true for schizophrenia in which the increased concentration of dopamine is known to be responsible for characteristic positive symptoms of disease (hallucinations and delusions) [33]. Toxoplasmosis is, however, suspected to play a role in other mental health disorders like Parkinson’s disease, obsessive-compulsive disorder, cryptic epilepsy, suicide, and unipolar depression, for review see [7]. Results of the present study indicate that the infection of *Bartonella*, rather than *Toxoplasma* is a more likely cause of unipolar depression and subclinical depression in women who were bitten or scratched by a cat.

## Limitations

Cross-sectional study is the most powerful and economic technique for demonstrating the existence of associations between two factors. It should be reminded, however, that no cross-sectional study could solve the question what is the cause and what the effect. It is theoretically possible that either depressed people are more often scratched (not bitten) by a cat or that cat-scratching results (in predisposed subjects) in depression.

Our population does not represent a typical sample of Czech and Slovak populations. Therefore, the data on prevalence of toxoplasmosis, incidences of animals-related injuries or prevalence of unipolar depression cannot be generalized to normal Czech and Slovak population. However, there are no reasons for expecting that, for example, the cat-related injuries influence the risk of unipolar depression only in this specific sample.

The most important limitation (but also strength, see below) of present study is that subjects self-reported their own *Toxoplasma* seropositivity/negativity status. It is certain that in some cases this information was wrong or obsolete. Latent toxoplasmosis is a lifelong disease; therefore, *Toxoplasma*-infected status does not change over time. However, some subjects that reported to be seronegative in the questionnaire acquired the infection during the time that passed from their serological examination. Moreover, even the best current serological methods have less than 100 % sensitivity. Published results suggest that in young subjects tested recently by IgG ELISA, about 5-10 % of seronegative subjects are in fact infected with *Toxoplasma* [34, 35]. Also, the results of the study performed on the population of 3,250 Czech male soldiers show that positivity to negativity seroconversions occur frequently in men after the age of 36 [19]. All these facts suggest that the epidemiological studies based on reported results of passed serological tests are more precise and sensitive than the cross-sectional studies which relay on the serological examination of current seropositivity/seronegativity status of participants. It should be also reminded that the existence of subjects with false negative results of serological tests cannot cause a false positive result in any study; however, it might cause its false negative result. We did not detected a significant association of toxoplasmosis with depression in our complex ANCOVA model. The absence of this effect persuaded us that cat scratching, rather than toxoplasmosis, could be responsible for the increased depression of animal-injured subjects. However, the absence of a statistical effect of toxoplasmosis could be also caused by the presence of false negatives in the population under study.

Another very important limitation of the present study is that the *Bartonella* negativity/positivity status of our subjects was not known. Unfortunately, available tests for *Bartonella* infection have rather low sensitivity and specificity and are only very rarely performed [36]. Our study should be performed in a country where medical records of the whole population are available for medical research.

## Conclusions

The results accumulated during past 20 years suggest that many behavioral and neurological changes, and probably also many mental health disorders, could be caused by infection with pathogens from a cat. If pathogens are truly involved it will be critical to know which pathogen is responsible for which disorder. No effective method of treatment of latent toxoplasmosis is currently available and the same is true also for bartonellosis [22]. It would, however, be useful to know what epidemiological measures could be effective and whether we have to search primarily for new antibiotics or new antiparasitic drugs.
